# Supplementary material for: Focal adhesions are controlled by microtubules through local contractility regulation
Source: EMBO J. 2024 May 20;43(13):9. doi: 10.1038/s44318-024-00114-4 (PMC11217342; doi:10.1038/s44318-024-00114-4)
Supplement: Supplementary file 1 — Appendix [file 44318_2024_114_MOESM1_ESM.pdf]

Appendix

Appendix figures (Page 2)

Appendix Figure S1 (Page 2)
Appendix Figure S2 (Page 4)
Appendix Figure S3 (Page 5)
Appendix Figure S4 (Page 6)
Appendix Figure S5 (Page 7)
Appendix Figure S6 (Page 8)

Appendix details of computational model (Page 9)

Appendix OptoKANK plasmids information (Page 15)

Appendix references (Page 20)

Appendix Figure

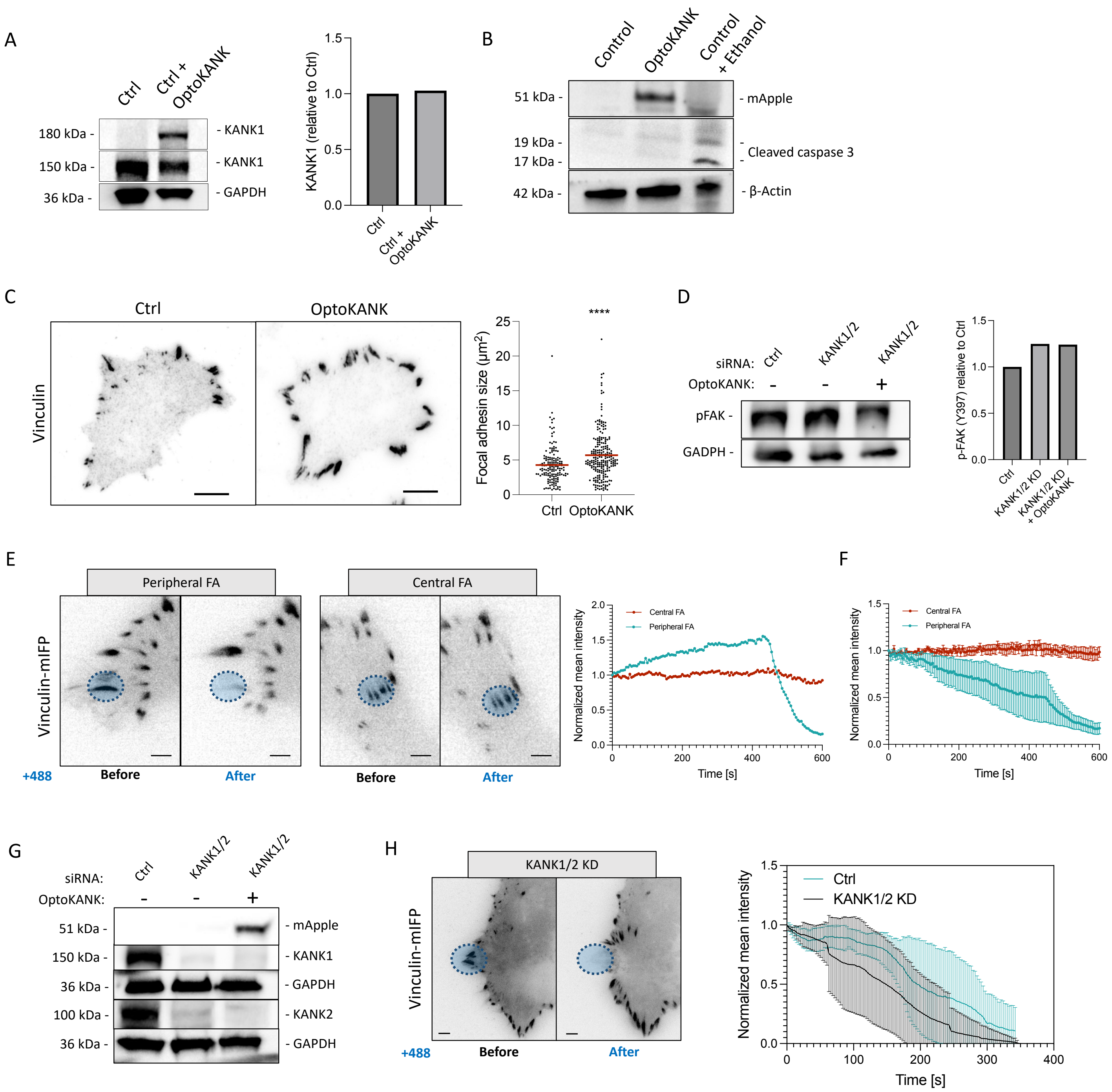

Appendix Figure S1

(A) Western Blots showing Control vs OptoKANK transfected HT1080 cells and probed for endogenous KANK1 (anti-KANK1), exogenous KANK1 (SSpB-ΔKN-mEmerald with anti-KANK1), and GAPDH loading control. The graph shows the KANK1 blot densitometries in control and exogenous KANK1 in OptoKANK transfected cells, normalized to GAPDH and relative to control.

(B) Western blots showing Control, OptoKANK transfected HT1080 cells and positive control (ethanol treated HT1080 cells) and probed for endogenous KANK1, mApple-KN-LOV2-ssrA and β-actin loading control and an indicator of cell death Cleaved Caspase 3.

(C) Representative images of HT1080 cells in control and after transfection with OptoKANK and stained for Vinculin. Graph shows the focal adhesion sizes in these conditions (**** p<0,0001 t-test, n = 18 cells for KANK1/2, 10 cells for Ctrl, both from 3 independent experiments; scale bar 10 µm).

(D) Western Blots showing Control, KANK1/2-depleted HT1080 cells and KANK1/2-depleted HT1080 cells transfected with OptoKANK and probed for p-FAK (Y397) and GAPDH loading control. The graph shows the p-FAK blot densitometries in these conditions, normalized to GAPDH and relative to control.

(E) Representative images of HT1080 cells in which OptoKANK has been activated (blue dotted line) in central focal adhesion vs peripheral focal adhesion with the corresponding vinculin intensity on the right graph (scale bar 5 µm).

(F) Graph shows the normalized mean vinculin intensity of illuminated focal adhesions on central vs peripheral focal adhesions over the time in these two conditions (mean ± s.e.m; n = 5 cells).

(G) Immunoblots of mApple, KANK1, KANK2 and GAPDH loading controls in control (Ctrl), in KANK1/2-depleted HT1080, and in KANK1/2-depleted HT1080 carrying the OptoKANK constructs.

(H) Representative images of Vinculin-mIFP-transfected HT1080 cells, depleted for KANK1/2 and carrying the OptoKANK constructs before and after blue light illumination on the encircled focal adhesion (blue dotted line. Graph shows the normalized mean vinculin intensity of illuminated focal adhesions over the time (Data are presented as mean ± s.e.m; n = 20 cells minimum from three independent experiments; p<0,0001 between Ctrl and KANK 1/2 KD; two-way ANOVA test. scale bar 5 µm).

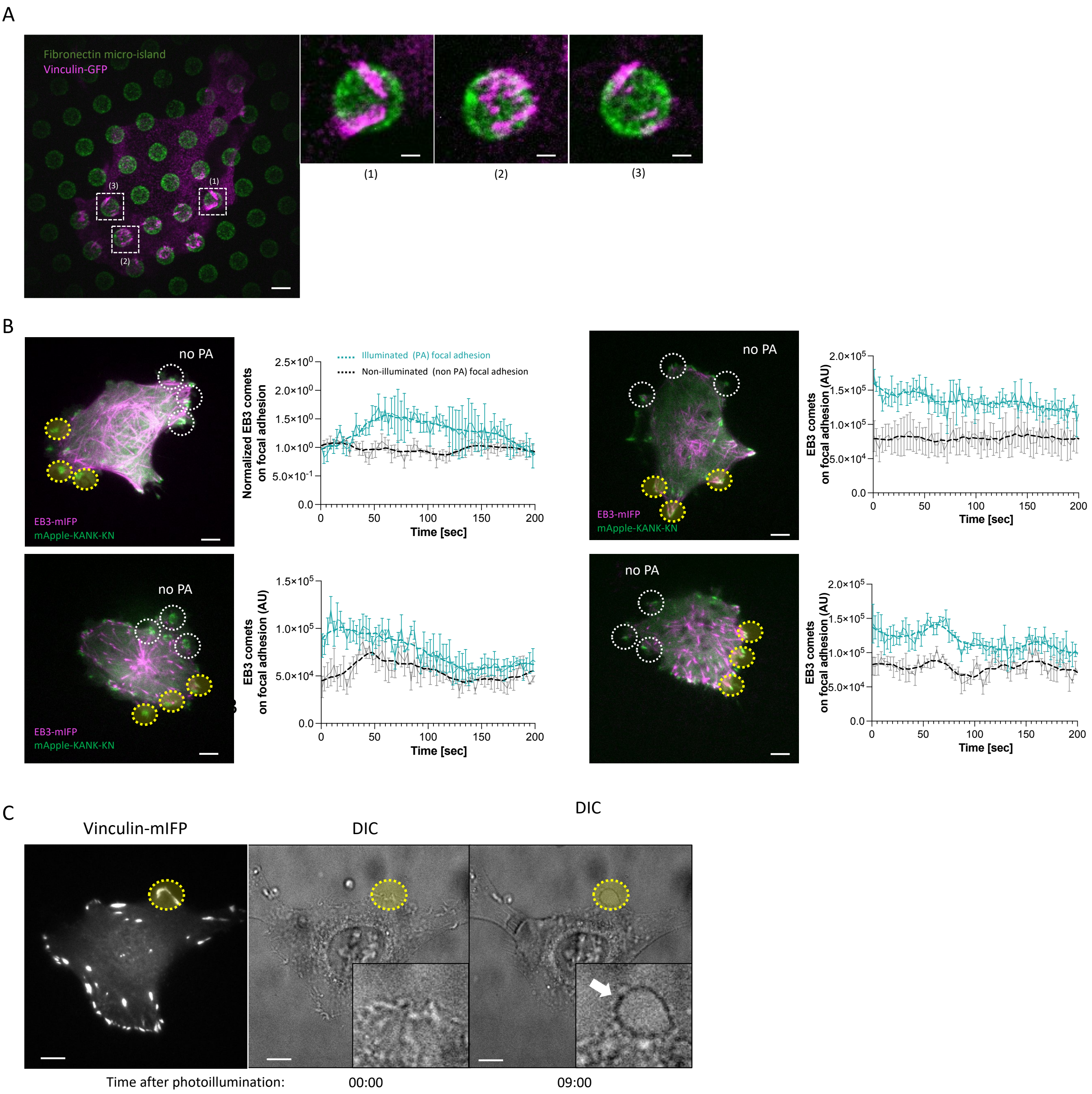

Appendix Figure S2

(A) Example of HT1080 cell transfected with vinculin-GFP, plated on microislands pattern labelled in far red (Alexa 647). Typically, the cell displays 2 to 4 focal adhesions par island where the microtubules visualized thanks to TAU protein (yellow) are passing by the focal adhesion. (Scale bar 5 µm)

(B) Graphs show the integrated EB3 comet fluorescence after processing with U-Track2 for photoactivated (yellow dotted line) and non-photoactivated (white dotted line) focal adhesions over the time. (Data are presented as the mean ± s.e.m of integrated EB3 fluorescence; n= 3 focal adhesions per cell; scale bar 5 µm). See also movie EV4.

(C) Representative images of Vinculin-mIFP-transfected HT1080 cells carrying the OptoKANK constructs after treatment with dynasore. Images show the illuminated focal adhesion (yellow dotted circle) using

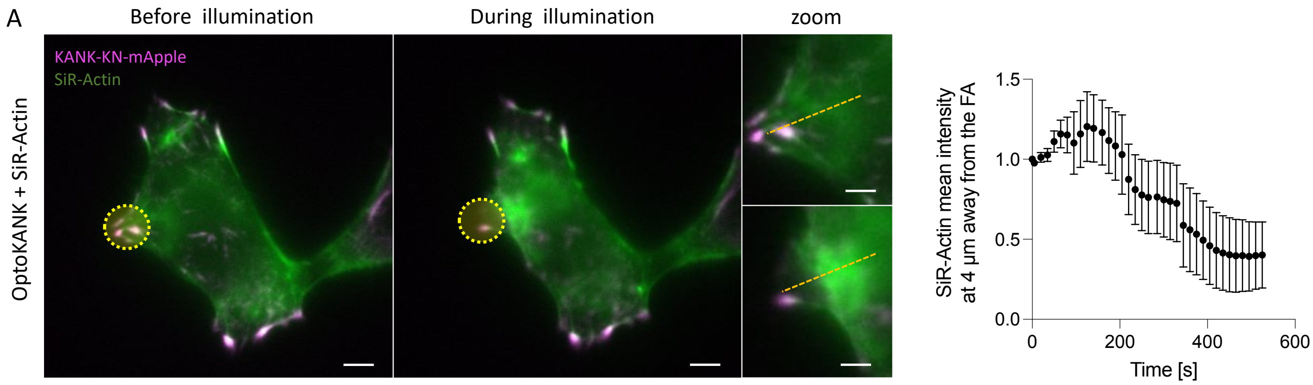

vinculin-mIFP staining and the DIC images showing the bleb formation (see white arrow in the box) after 9 min of OptoKANK activation (scale bar 10 µm). See also movie EV6.

Appendix Figure S3

Representative image of OptoKANK-transfected HT1080 cell before and during OptoKANK activation of the selected focal adhesion (yellow dotted line). SiR-Actin was used to assess the myosin-II dynamics upon OptoKANK activation. The line scan was used to measure the SiR-Actin intensity in the vicinity (4 µm away in centripetal direction) of the proximal end of the photoactivated focal adhesion shown. The graph shows the SiR-Actin mean intensity at this distance upon OptoKANK activation (Data are presented as mean ± s.e.m; n = 7 cells; scale bar 5 µm)). See also movie EV8.

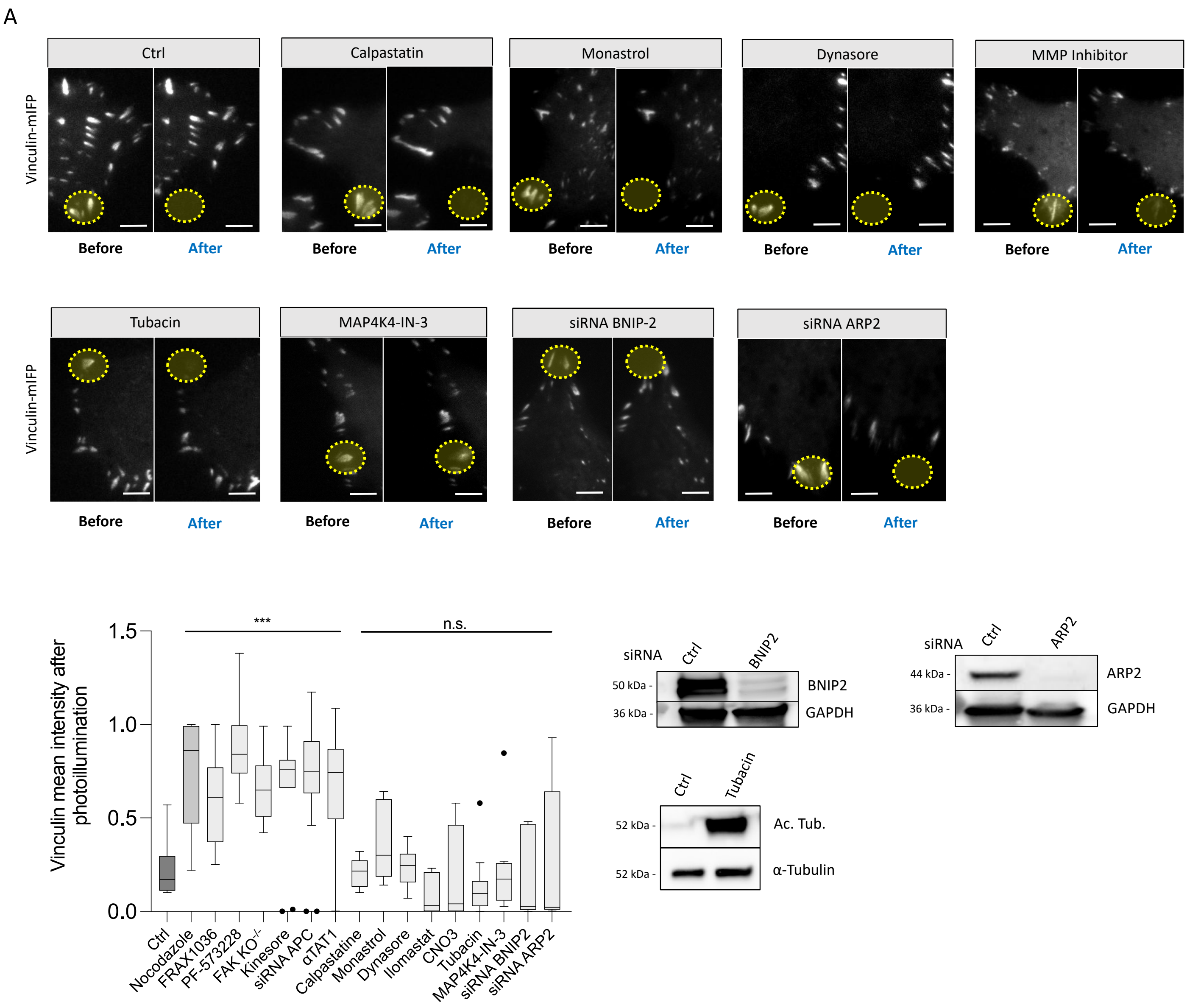

Appendix Figure S4

Representative images of Vinculin-mIFP-transfected HT1080 cells carrying the OptoKANK constructs before and after blue light illumination of the focal adhesion (yellow dotted line) for control cells, cells treated with a calpain inhibitor, calpastatin, a kinesin-V inhibitor, monastrol, a dynamic inhibitor, dynasore, a MMP inhibitor, Ilomastat, a MAP4K4 inhibitor, MAP4K4-IN-3, and depleted for BNIP2 and ARP2. Graph shows the normalized mean vinculin intensity after the illumination of cells treated as indicated (Data are presented as mean ± s.e.m; Ctrl, n =18 cells; Nocodazole, n = 12 cells; FRAX1036, n = 15 cells; PF-573228, n = 10 cells; FAK KO$^{-/-}$, n = 8 cells; Kinesore, n = 11 cells; siRNA APC, n = 18 cells; αTAT1, n = 10 cells; n = 12 cells; Calpastatin, n = 10 cells; Monastrol, n = 8 cells; Dynasore, n = 10 cells; MMP inhibitor, n = 7 cells ; CNO3, n = 8 cells; Tubacin, n = 12 cells, MAP4K4-IN-3, n = 9 cells; siRNA BNIP2, n = 16 cells; siRNA ARP2, n = 10 cells; one-way ANOVA, *** p < 0,001, N.S. not significant; the center line denotes the median value (50th percentile) while the box contains the 25th to 75th percentiles of dataset. The black whiskers mark the minimum and maximum percentiles, and values beyond these upper and lower bounds are considered outliers; scale bar 5 µm). Immunoblots of BNIP2, ARP2, acetylated tubulin and GAPDH are shown in the black box.

A

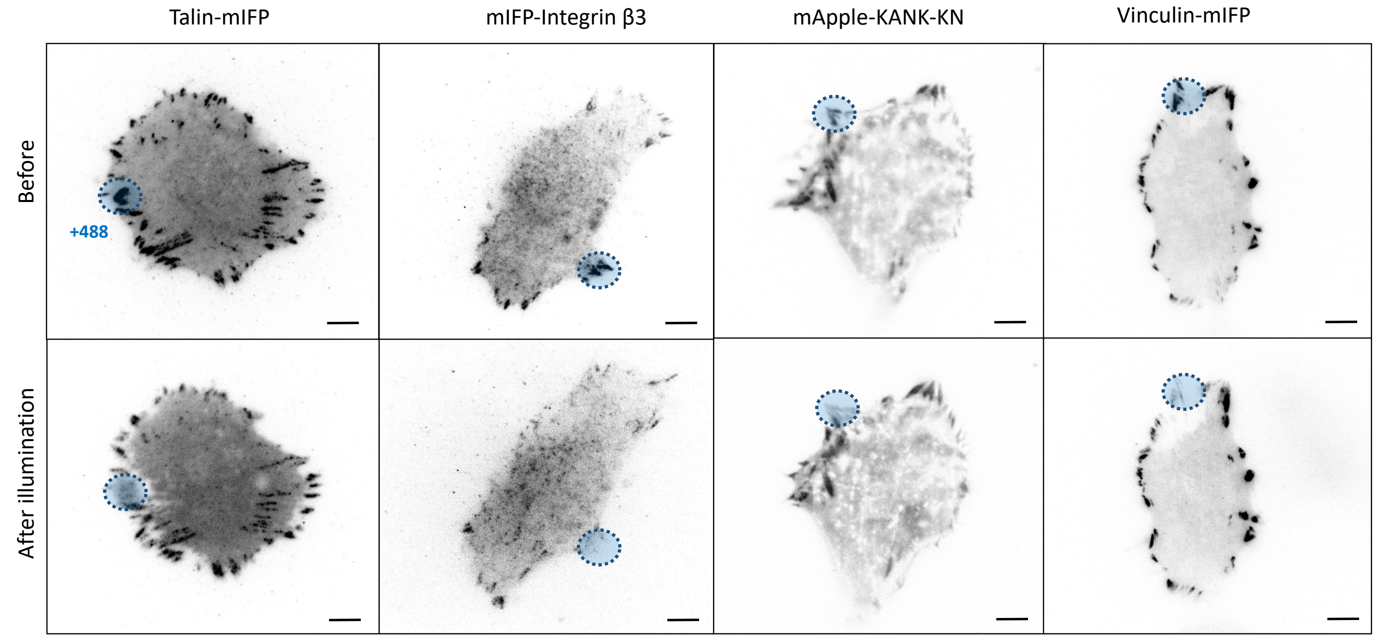

B

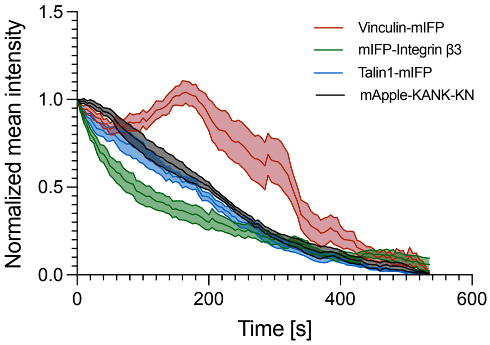

Appendix Figure S5

(A) Representative images of HT1080 cells carrying the OptoKANK constructs before and after blue light illumination of areas (blue dotted line) containing focal adhesion visualized using Talin-mIFP, mIFP-Integrin β3, mApple-KANK-KN and Vinculin-mIFP labeling (scale bar 10 μm).

(B) Normalized mean intensity of Vinculin (vinculin-mIFP), Integrin β3 (mIFP-Integrin β3), Talin1 (Talin1-mIFP) and KN (mApple-KANK-KN) over the illuminated focal adhesion of cells carrying OptoKANK.

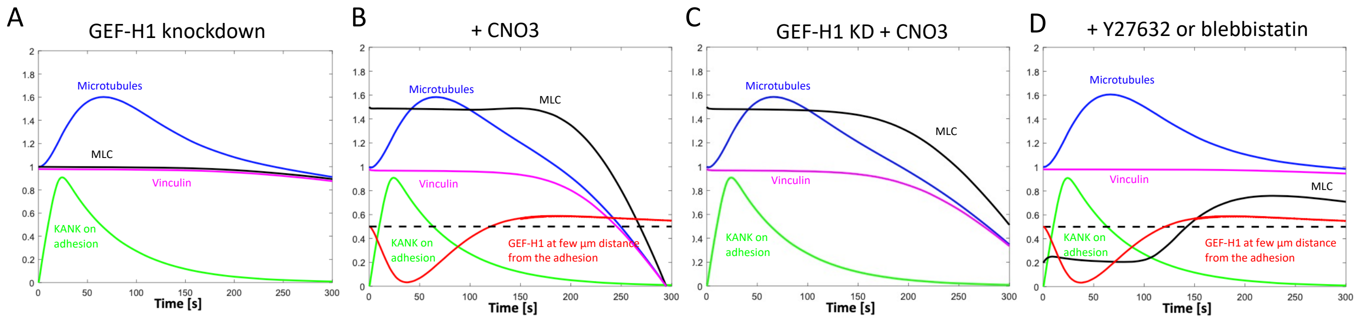

Appendix Figure S6

Model-predicted time series for microtubule, vinculin, KANK, GEF-H1 and myosin densities for the following simulated perturbations: GEF-H1 knockdown **(A)**, treatment with CNO3 **(B)**, GEF-H1 knockdown + CNO3 **(C)** and treatment with Y27632 or blebbistatin **(D)**. Model parameters used in the simulations are described in the Supplemental Text.

Appendix details of computational model

The conceptual model is shown schematically in **Figure 6**. Model variables and parameters are listed in Supplemental Tables 1 and 2, respectively. Below, we discuss the dimensions and parameter values in detail. We discuss the model assumptions along with describing mathematical terms in the model equations. The first three model equations introduce the dynamics of the number of microtubules (MTs) on the focal adhesion (FA), number of KANK molecules on the FA, and active myosin density proximal to the FA, respectively:

$$\frac{dT}{dt} = 1 - c\exp(-K)T \qquad (1)$$

$$\frac{dK}{dt} = i(t) - kH(T-1)K \qquad (2)$$

$$\frac{dM}{dt} = m_1 + m_2 f_1(G) - m_3 M \qquad (3)$$

Here, T is the number of MT plus ends on the FA, K is the number of KANK molecules on the FA, and M is the active myosin density proximal to the FA. The model does not describe explicit spatial molecular distributions, and so we only follow the temporal dynamics. Furthermore, even though MT and KANK numbers are not large, we approximate these numbers with continuous variables and from here on calling them 'densities' (these densities can be thought of as the respective numbers divided by the FA area). Also, we neglect stochasticity of these numbers and consider deterministic continuous model. Lastly, all densities in the model are non-dimensional, measured in units of characteristic observed scales. In the conceptual model, in the absence of the data on density dependence of relevant chemical rates, the dimensional numbers for the variables are not crucial.

Left-hand-sides of Equations 1-3 are the rates of change of the respective variables; we now turn to description of the right-hand-sides' terms. The first term in Equation 1 describes arrival of the polymerizing growing MTs at a constant rate at the FA. This non-dimensional rate is chosen to be equal to unity because of the scaling discussed in the previous paragraph. The constancy of this rate is reasonable based on our observations suggesting that near the cell margins, the overall MT number and average dynamic instability parameters away from but proximal to adhesions do not exhibit noticeable variations. The second term is responsible for MTs leaving the FA with the rate equal to $c\exp(-K)$, where c is the inverse pause time for MT prior to MT disassembly, before KANK activation. This parameter value can be estimated based on the observation of the MT pauses on the order of tens of seconds on the FAs (Azoitei et al 2019). The factor $\exp(-K)$ is responsible for the increase of the MT pause on the FA (and so the rate of leaving decrease) with growing KANK

number (Bouchet2016). The exact functional form of this dependence is unknown, and the exponential form we use is a reasonable assumption. Implicitly, we use another assumption – that this rate decreases a few-fold when KANK is fully loaded onto the FA upon the illumination – which is based on the observed 1.5-fold increase of the MT number on the FA after the illumination. Note also that in principle there could be an effect of the MT catastrophe being promoted by the attachment of microtubule to the FA (Efimov and Kaverina 2009), but this effect can be effectively accounted for by the constant parameter c.

In Equation 2, the first term describes loading of KANK to the FA upon the illumination, as the following function of time:

$$i(t) = K_1 \frac{\exp\left(s_1\left(t_0 - t\right)\right)}{1 + \exp\left(s_1\left(t_0 - t\right)\right)} \qquad (4)$$

This function is a smoothed step function equal to constant, K_1, before time t_0, and zero after this time. Parameter K_1 (Supplemental Table 2) is chosen so that by the end of the loading, KANK density reaches a characteristic unit; for t_0 (Supplemental Table 2), we use the observed time of 20-30 seconds; smoothing parameter s_1 (Supplemental Table 2) is chosen to account for a sharp step-function-like transition. The second term in this equation accounts for the observed dissociation of KANK molecules from the FA on the scale of tens of seconds (constant parameter k (Supplemental Table 2)). We also assume that MTs bring with them an adhesion-weakening molecule (Yue et al 2014), which triggers dissociation of integrins and talins, and because KANK binds to talin, of KANK molecules. This effect is accounted for by the factor $H\left(T-1\right)$, where H is Heaviside function equal to zero when the MT density is less or equal to unity, and to one otherwise. Thus, we assume that this effect has a threshold character. Another possibility is that KANK proteins themselves, without the MTs, diminish the talin-actomyosin linkage, which curbs force transmission across integrins, leading to reduced integrin–ligand bond strength, slippage between integrin and ligand, and sliding (Sun et al 2016). In that case, the factor $H\left(T-1\right)$ must be absent from Equation 2, and instead the dissociation rate simply equals constant k. The model's results compare to the observations equally well with both choices.

In Equation 3, the first term is the constant (m_1 (Supplemental Table 2)) basal, GEF-H1-independent activation rate of myosin, and the third term is the respective deactivation rate (which is assumed to be the first order chemical reaction with rate m_3 (Supplemental Table 2)). The constant m_1 is chosen

to bring the basal active myosin density to unity; the constant m_3 is chosen based on MLC phosphorylation cycle on the order of tens of seconds (Amano et al 1996). Note that in order to not overwhelm the model with molecular complexity, we do not explicitly describe the intermediate steps, like action of RhoGTPases, activation of ROCK, which are relatively fast, on the order of seconds (Bolado-Carrancio et al 2020) and can be lumped together with MLC phosphorylation rate. We also do not explicitly describe myosin clusters' assembly. In the model, the myosin density is assumed to be proportional to the effective traction force and is a harbinger of the observed pMLC density.

The second term in Equation 3 is the GEF-H1-dependent rate of myosin activation. Rate m_2 (Supplemental Table 2) characterizes the relative effect of GEF-H1-dependent compared to GEF-H1-independent rate. This parameter is unknown and chosen to fit the data. Function $f_1(G)$ accounts for assumed threshold character of the GEF-H1 action:

$$f_1(G) = \frac{\exp\left(s_2\left(G-G_0\right)\right)}{1+\exp\left(s_2\left(G-G_0\right)\right)} \qquad (5)$$

Here G_0 is the characteristic threshold density of activated GEF-H1 in the cytoplasm in the vicinity of the assembling actomyosin array proximal to the FA. Below this threshold, there is no effect, above the threshold there is a saturated effect. The threshold value is unknown and is chosen to fit the data (Supplemental Table 2). Parameter s_2 (Supplemental Table 2) characterizes the sharpness of the threshold effect; it is unknown from the experiment and is chosen to fit the data.

To close the system of model equations, we need GEF-H1 density as a function of time. The following equation provides such density at distance L from the FA:

$$G(L,t) = G_0 - g\int_0^t \frac{dT}{dt}(\tau)\times\frac{\exp\left(-L^2/\left(4D(t-\tau)\right)\right)}{\sqrt{4\pi D(t-\tau)}}\times d\tau \quad (6)$$

This formula is based on the following assumptions and estimates. First, MTs are effectively a sink for GEF-H1 molecules (Krendel2002). Thus, we assume that any time a MT arrives at the FA, a certain number of GEF-H1 molecules, equal to g, is locally absorbed from the cytoplasm. The number of MTs arriving per time Δt is $\frac{dT}{dt}\Delta t$, and so $g\,\frac{dT}{dt}\Delta t$ molecules are absorbed during this time interval. This expression assumes that the rate of absorption is independent of the local GEF-H1 concentration in the cytoplasm, so the limiting factor in the absorption is the local MT

length/number and not GEF-H1 diffusion. Value of parameter g (Supplemental Table 2) is unknown and chosen to fit the data. Similarly, if MTs are leaving the FA but not arriving, $g\ \frac{dT}{dt}\Delta t$ molecules are released during this time interval, assuming that GEF-H1 molecules are released from tubulin instantly upon the MT disassembly, so there is then a local source of GEF-H1 molecules on the FA. Neglecting local sinks and sources elsewhere in the vicinity, the spatially explicit concentration of GEF-H1 molecules in the cytoplasm can be described by the following reaction-diffusion equation:

$$\frac{\partial G}{\partial t}=\frac{dT}{dt}\delta\left(x\right)+D\Delta G$$

Here $\delta\left(x\right)$ is the delta-function responsible for the location of the source/sink at the FA location, Δ is the Laplacian operator, and D is the diffusion coefficient of GEF-H1 in the cytoplasm. We use a one-dimensional approximation of the geometry where x becomes the distance inward from the cell margin. In this approximation, the exact analytical solution for this reaction-diffusion equation is Equation 6 (Strauss 2007), where now L is the distance between the FA and proximal actomyosin array. Parameter L (Supplemental Table 2), based on our data is several microns. Value of parameter D (Supplemental Table 2), on the order of square microns per second, can be indirectly estimated from the data reported in (Azoitei et al 2019). To not overwhelm the model with molecular complexity, we do not explicitly include the step of activation of the released GEF-H1 molecules (Azoitei et al 2019), however, such process will not qualitatively change the conclusions from the model. One possible variant of the model is that we can in principle include the acetylation of MTs at the FA, and subsequent release of GEF-H1 from the MTs independent of the MT disassembly, since GEF-H1 has a low affinity to the acetylated MTs (Seetharaman et al 2022). Such variant of the model will lead to the same conclusions as our basic model.

Lastly, the mechanics of the FA slippage is accounted for in the model as follows. We assume that the pulling force applied to the FA, and therefore the traction force is directly proportional to the myosin density in the model. Next, we assume that the FA is 'gripping' if the ratio of the pulling force to adhesive strength is below a threshold, and slipping, if this ratio is above the threshold. We assume that the adhesive strength is proportional to the KANK density on the FA (not that KANK directly contributes to the adhesive strength, but rather that its adhesive molecular partners do, and their numbers are proportional to the KANK numbers), and that there is some basal, KANK-independent part of the strength. Mathematically, this translates into the assumption that the adhesive strength is proportional to the expression $\left(K_0+K\right)$, where K_0 is a constant parameter.

Then, the ratio of the pulling force to adhesive strength is proportional to the expression $\left(M/\left(K_0+K\right)\right)$, and we define the velocity of slippage as follows:

$$V = V_0 f_2\left(M/\left(K_0+K\right)\right) \qquad (7)$$

Here V_0 is the characteristic magnitude of the slippage velocity (Supplemental Table 2) that can be estimated from our data. Threshold function f_2 is defined as follows:

$$f_2\left(f\right) = \frac{\exp\left(s_3\left(f-f_0\right)\right)}{1+\exp\left(s_3\left(f-f_0\right)\right)} \qquad (8)$$

Here f_0 is the threshold force to adhesion strength ratio and s_3 is the sharpness of the threshold parameter. The values of parameters K_0, f_0 and s_3 are unknown and chosen to fit the data (the results are insensitive to the value of s_3). The shift of the FA due to the slipping is given by the formula:

$$X = \int_0^t V\left(\tau\right) d\tau \qquad (9)$$

There is a gradual physical slipping of MTs, KANK and myosin, together with the slipping FA, and so we assume that the measured MT, KANK and myosin densities on the area of the initial adhesion scale with the initial adhesion length, on the order of one micron, minus the shift. Therefore, when the shift becomes equal to the initial adhesion length, the measured densities approach zero. Thus, as a result, we plot expressions $\left(1-X\right)\times T, \left(1-X\right)\times M$ for the measured MT and myosin density. KANK density decreases so significantly by the time the slippage starts, that factor $\left(1-X\right)$ does not affect the result. Finally, we assume that the measured vinculin density is effectively a marker for the slipping adhesion area and approximate the measured vinculin density with expression $A = 1-X$.

Results: The model equations are integrated by using standard Euler numerical scheme. The results are shown in Figure 6F. Here is the qualitative explanation for the predicted molecular densities as functions of time. KANK is rapidly loaded on the FA upon illumination, and then, as a part of the gradual, MT-induced weakening of the FA, starts to decrease exponentially. The transient accumulation of KANK causes longer pauses of the MTs on the FA, so the MT density on the FA increases at first, up to ~ 1.5-fold its value before the illumination, but after ~ 60 sec starts to decrease because the diminishing KANK number leads to shorter MT pauses on the FA.

Table S1: model variables

T	Number of microtubule tips on the adhesion
K	Number of KANK molecules on the adhesion
M	Number of activated MLCs proximal to the adhesion
G	Activated GEF concentration proximal to the adhesion
V	Rate of slippage of the adhesion
X	Displacement of the adhesion
A	Number of vinculin molecules in the adhesion

Table S2: model parameters

c	Rate of microtubules' leaving the adhesion	1/30 sec
k	Rate of KANK dissociation	1/60 sec
m_1	Basal rate of MLC activation	1 (control,GEFKD), 1.5 (CNO) 0.2 (BlebY)
m_2	GEF-H1-dependent rate of MLC activation	5 (control), 0.2 (BlebY), 0.1 (CNO), 0 (GEFKD)
m_3	Basal rate of MLC deactivation	1/30 sec
G_0	Basal GEF-H1 concentration in the cytoplasm	0.5
g	Sequestered/released GEF-H1 amount per microtubule	0.01
L	Distance between the adhesion and myosin assembly site	4 μm
D	GEF-H1 diffusion coefficient in the cytoplasm	1 μm^2/sec
V_0	Characteristic slippage velocity	0.5 μm/sec
K_0	Basal strength of a weak adhesion	0.2
f_0	Slippage force/adhesion ratio	7.5
K_1	Rate of KANK-adhesion association upon illumination	2.2
t_0	Characteristic time of KANK loading upon illumination	20 sec
s_1	Inverse threshold width for KANK loading function	0.1
s_2	Inverse threshold width for GEF-H1-dependent rate of MLC activation	0.05
s_3	Inverse threshold width for force-dependent rate of adhesion slippage	1

Appendix OptoKANK plasmids information

pmApple-KN-LOV2ssrA

```
ORIGIN
        1 tagttattaa tagtaatcaa ttacggggtc attagttcat agcccatata tggagttccg
       61 cgttacataa cttacggtaa atggcccgcc tggctgaccg cccaacgacc cccgcccatt
      121 gacgtcaata atgacgtatg ttcccatagt aacgccaata gggactttcc attgacgtca
      181 atgggtggag tatttacggt aaactgccca cttggcagta catcaagtgt atcatatgcc
      241 aagtacgccc cctattgacg tcaatgacgg taaatggccc gcctggcatt atgcccagta
      301 catgacctta tgggactttc ctacttggca gtacatctac gtattagtca tcgctattac
      361 catggtgatg cggttttggc agtacatcaa tgggcgtgga tagcggtttg actcacgggg
      421 atttccaagt ctccacccca ttgacgtcaa tgggagtttg ttttggcacc aaaatcaacg
      481 ggactttcca aaatgtcgta acaactccgc cccattgacg caaatgggcg gtaggcgtgt
      541 acggtgggag gtctatataa gcagagctgg tttagtgaac cgtcagatcc gctagccacc
      601 atggtgagca agggcgagga gaataacatg gccatcatca aggagttcat gcgcttcaag
      661 gtgcacatgg agggctccgt gaacggccac gagttcgaga tcgagggcga gggcgagggc
      721 cgcccctacg aggcctttca gaccgctaag ctgaaggtga ccaagggtgg ccccctgccc
      781 ttcgcctggg acatcctgtc ccctcagttc atgtacggct ccaaggtcta cattaagcac
      841 ccagccgaca tccccgacta cttcaagctg tccttccccg agggcttcag gtgggagcgc
      901 gtgatgaact tcgaggacgg cggcattatt cacgttaacc aggactcctc cctgcaggac
      961 ggcgtgttca tctacaaggt gaagctgcgc ggcaccaact tcccctccga cggccccgta
     1021 atgcagaaga agaccatggg ctgggaggcc tccgaggagc ggatgtaccc cgaggacggc
     1081 gccctgaaga gcgagatcaa gaagaggctg aagctgaagg acggcggcca ctacgccgcc
     1141 gaggtcaaga ccacctacaa ggccaagaag cccgtgcagc tgcccggcgc ctacatcgtc
     1201 gacatcaagt tggacatcgt gtcccacaac gaggactaca ccatcgtgga acagtacgaa
     1261 cgcgccgagg gccgccactc caccggcggc atggacgagc tgtacaaggt cgacctcgag
     1321 atggctcaca ccacaaaggt taacggcagt gcctcaggaa aagcaggtga tattctcagt
     1381 ggagaccagg acaaggaaca gaaagaccct tactttgtgg agacccccta tggttatcaa
     1441 ctagacttag atttcctcaa atatgtggat gacatacaga agggaaatac catcaaaaga
     1501 ctgaacatcc agaagaggcg gaaggaattc accggttctg gatccgggga gtttctggca
     1561 accacactgg aacggatcga gaaaaatttc gtgattactg atccgagact gcctgacaac
     1621 ccaatcattt ttgcgagcga ttccttcctg cagctgacag aatattctcg ggaagagatc
     1681 ctggggcgca attgccgttt tctgcaggga cccgagacag accgtgccac tgttcggaaa
     1741 atcagagatg ctattgacaa ccagactgaa gtgaccgttc agctgatcaa ttataccaag
     1801 agcggcaaga agttctggaa cgtgttccac ctgcagccga tgcgcgatta taagggcgac
     1861 gtccagtact tcattggcgt gcagctggat ggcaccgaac gtcttcatgg cgccgctgag
     1921 cgtgaggcgg tctgcctgat caaaaagaca gcctttcaga ttgctgaggc agcgaacgac
     1981 gaaaattact tttaagcggc cgcgactcta gatcataatc agccatacca catttgtaga
     2041 ggttttactt gctttaaaaa acctcccaca cctccccctg aacctgaaac ataaaatgaa
     2101 tgcaattgtt gttgttaact tgtttattgc agcttataat ggttacaaat aaagcaatag
     2161 catcacaaat ttcacaaata aagcattttt ttcactgcat tctagttgtg gtttgtccaa
     2221 actcatcaat gtatcttaag gcgtaaattg taagcgttaa tattttgtta aaattcgcgt
     2281 taaatttttg ttaaatcagc tcatttttta accaataggc cgaaatcggc aaaatccctt
     2341 ataaatcaaa agaatagacc gagatagggt tgagtgttgt tccagtttgg aacaagagtc
     2401 cactattaaa gaacgtggac tccaacgtca aagggcgaaa aaccgtctat cagggcgatg
     2461 gcccactacg tgaaccatca ccctaatcaa gttttttggg gtcgaggtgc cgtaaagcac
     2521 taaatcggaa ccctaaaggg agcccccgat ttagagcttg acggggaaag ccggcgaacg
     2581 tggcgagaaa ggaagggaag aaagcgaaag gagcgggcgc tagggcgctg gcaagtgtag
     2641 cggtcacgct gcgcgtaacc accacacccg ccgcgcttaa tgcgccgcta cagggcgcgt
     2701 caggtggcac ttttcgggga aatgtgcgcg gaacccctat ttgtttattt ttctaaatac
     2761 attcaaatat gtatccgctc atgagacaat aaccctgata aatgcttcaa taatattgaa
     2821 aaaggaagag tcctgaggcg gaaagaacca gctgtggaat gtgtgtcagt tagggtgtgg
     2881 aaagtcccca ggctccccag caggcagaag tatgcaaagc atgcatctca attagtcagc
     2941 aaccaggtgt ggaaagtccc caggctcccc agcaggcaga agtatgcaaa gcatgcatct
     3001 caattagtca gcaaccatag tcccgcccct aactccgccc atcccgcccc taactccgcc
     3061 cagttccgcc cattctccgc cccatggctg actaattttt tttatttatg cagaggccga
     3121 ggccgcctcg gcctctgagc tattccagaa gtagtgagga ggcttttttg gaggcctagg
     3181 cttttgcaaa gatcgatcaa gagacaggat gaggatcgtt tcgcatgatt gaacaagatg
     3241 gattgcacgc aggttctccg gccgcttggg tggagaggct attcggctat gactgggcac
     3301 aacagacaat cggctgctct gatgccgccg tgttccggct gtcagcgcag gggcgcccgg
     3361 ttctttttgt caagaccgac ctgtccggtg ccctgaatga actgcaagac gaggcagcgc
     3421 ggctatcgtg gctggccacg acgggcgttc cttgcgcagc tgtgctcgac gttgtcactg
```

```
     3481 aagcgggaag ggactggctg ctattgggcg aagtgccggg gcaggatctc ctgtcatctc
     3541 accttgctcc tgccgagaaa gtatccatca tggctgatgc aatgcggcgg ctgcatacgc
     3601 ttgatccggc tacctgccca ttcgaccacc aagcgaaaca tcgcatcgag cgagcacgta
     3661 ctcggatgga agccggtctt gtcgatcagg atgatctgga cgaagagcat caggggctcg
     3721 cgccagccga actgttcgcc aggctcaagg cgagcatgcc cgacggcgag gatctcgtcg
     3781 tgacccatgg cgatgcctgc ttgccgaata tcatggtgga aaatggccgc ttttctggat
     3841 tcatcgactg tggccggctg ggtgtggcgg accgctatca ggacatagcg ttggctaccc
     3901 gtgatattgc tgaagagctt ggcggcgaat gggctgaccg cttcctcgtg ctttacggta
     3961 tcgccgctcc cgattcgcag cgcatcgcct tctatcgcct tcttgacgag ttcttctgag
     4021 cgggactctg gggttcgaaa tgaccgacca agcgacgccc aacctgccat cacgagattt
     4081 cgattccacc gccgccttct atgaaaggtt gggcttcgga atcgttttcc gggacgccgg
     4141 ctggatgatc ctccagcgcg gggatctcat gctggagttc ttcgcccacc ctagggggag
     4201 gctaactgaa acacggaagg agacaatacc ggaaggaacc cgcgctatga cggcaataaa
     4261 aagacagaat aaaacgcacg gtgttgggtc gtttgttcat aaacgcgggg ttcggtccca
     4321 gggctggcac tctgtcgata ccccaccgag accccattgg ggccaatacg cccgcgtttc
     4381 ttccttttcc ccaccccacc ccccaagttc gggtgaaggc ccagggctcg cagccaacgt
     4441 cggggcggca ggccctgcca tagcctcagg ttactcatat atactttaga ttgatttaaa
     4501 acttcatttt taatttaaaa ggatctaggt gaagatcctt tttgataatc tcatgaccaa
     4561 aatcccttaa cgtgagtttt cgttccactg agcgtcagac cccgtagaaa agatcaaagg
     4621 atcttcttga gatccttttt ttctgcgcgt aatctgctgc ttgcaaacaa aaaaaccacc
     4681 gctaccagcg gtggtttgtt tgccggatca agagctacca actctttttc cgaaggtaac
     4741 tggcttcagc agagcgcaga taccaaatac tgtccttcta gtgtagccgt agttaggcca
     4801 ccacttcaag aactctgtag caccgcctac atacctcgct ctgctaatcc tgttaccagt
     4861 ggctgctgcc agtggcgata agtcgtgtct taccgggttg gactcaagac gatagttacc
     4921 ggataaggcg cagcggtcgg gctgaacggg gggttcgtgc acacagccca gcttggagcg
     4981 aacgacctac accgaactga gatacctaca gcgtgagcta tgagaaagcg ccacgcttcc
     5041 cgaagggaga aaggcggaca ggtatccggt aagcggcagg gtcggaacag gagagcgcac
     5101 gagggagctt ccagggggaa acgcctggta tctttatagt cctgtcgggt ttcgccacct
     5161 ctgacttgag cgtcgatttt tgtgatgctc gtcagggggg cggagcctat ggaaaaacgc
     5221 cagcaacgcg gcctttttac ggttcctggc cttttgctgg ccttttgctc acatgttctt
     5281 tcctgcgtta tcccctgatt ctgtggataa ccgtattacc gccatgcat
//
```

SSpB-deltaKN (69-1352)-mEmerald

```
ORIGIN
        1 tagttattaa tagtaatcaa ttacggggtc attagttcat agcccatata tggagttccg
       61 cgttacataa cttacggtaa atggcccgcc tggctgaccg cccaacgacc cccgcccatt
      121 gacgtcaata atgacgtatg ttcccatagt aacgccaata gggactttcc attgacgtca
      181 atgggtggag tatttacggt aaactgccca cttggcagta catcaagtgt atcatatgcc
      241 aagtacgccc cctattgacg tcaatgacgg taaatggccc gcctggcatt atgcccagta
      301 catgacctta tgggactttc ctacttggca gtacatctac gtattagtca tcgctattac
      361 catggtgatg cggttttggc agtacatcaa tgggcgtgga tagcggtttg actcacgggg
      421 atttccaagt ctccacccca ttgacgtcaa tgggagtttg ttttggcacc aaaatcaacg
      481 ggactttcca aaatgtcgta acaactccgc cccattgacg caaatgggcg gtaggcgtgt
      541 acggtgggag gtctatataa gcagagctgg tttagtgaac cgtcagatcc gctagccacc
      601 atggaattca gctccccgaa acgccctaag ctgctgcgtg aatattacga ttggctggtt
      661 gataacagct ttaccccata tctggtggtg gatgccacat acctgggcgt gaacgtgccc
      721 gtggagtatg tgaaagacgg tcagatcgtg ctgaatctgt ctgcaagtgc gaccggcaac
      781 ctgcaactga caaatgattt tatccagttc aacgcccgct ttaagggcgt gtctcgtgaa
      841 ctgtatatcc cgatgggtgc cgctctggcc atttacgctc gcgagaacgg cgatggtgtg
      901 atgttcgaac cagaagaaat ctatgacgag ctgaatattg gtggtggttc tggtggtagc
      961 actagtgata tcccgtccgt gccatgccca gaacccagga ccacatctgg tcagcaaggt
     1021 atatggactt ccactgaatc cctctcatcc tccaacagtg atgacaacaa gcagtgcccc
     1081 aacttcctca tagccagaag tcaagttaca tcaactccaa tctcaaagcc acctccccct
     1141 ctggagacct cactcccttt tcttaccatc ccagaaaatc gacagctgcc acctccctca
     1201 ccacaactcc caaagcataa ccttcatgtc accaagacac tgatggagac ccggagaaga
     1261 ctggaacagg agagagccac catgcagatg acaccgggtg agttcagaag gcccaggctg
     1321 gccagttttg gaggcatggg caccacaagc tccctccctt cttttgtggg ttctggaaac
     1381 cacaagcctg ccaagcacca gcttcagaat ggataccaag gtaatgggga ttatggtagc
     1441 tatgccccag ctgctcccac cacttcctcc atggggagct ccatccgcca cagccccctg
     1501 agctcaggga tctccacccc agtgaccaac gtgagcccca tgcacctgca gcacatccgc
```

1561 gagcagatgg ccattgctct gaaacgcctg aaggagctgg aggagcaggt gcgaaccatc
1621 cctgtgctcc aggtaaagat ctctgtcttg caagaagaga aaaggcagtt ggtctcacag
1681 ctgaaaaacc aaagggctgc atcccagatc aatgtctgtg gtgtgaggaa gcggtcctat
1741 agtgcgggga acgcctccca gctggaacag ctctcccggg cccgaagaag tggcggggaa
1801 ttatacattg actatgagga ggaagaaatg gagaccgtag aacagagcac gcagaggata
1861 aaggagttcc ggcaacttac agcagacatg caagccctgg agcagaagat ccaggacagc
1921 agctgtgagg cctcctcaga gctcagggag aatggagagt gccggtctgt ggctgtgggt
1981 gccgaggaga acatgaacga catcgtcgtg taccacagag gctccaggtc ctgtaaggat
2041 gcagctgtag ggacacttgt tcagatgaga aattgtgggg tcagcgtgac agaggccatg
2101 cttggagtga tgactgaagc tgacaaagaa attgagctgc aacagcagac catagaatcc
2161 ttgaaggaaa agatctatcg cctagaagta cagcttagag aaaccaccca tgaccgggag
2221 atgactaaac tgaaacaaga gctgcaggct gctggatcga ggaaaaaggt tgacaaagcc
2281 acgatggccc agccgcttgt tttcagtaag gtggtggagg cagtggtgca gaccagagac
2341 caaatggtcg gcagtcacat ggacctggtg gacacgtgtg ttgggacctc cgtggaaaca
2401 aacagtgtag gcatctcctg ccagcctgaa tgtaagaata aagtcgtagg gcctgagctg
2461 cctatgaatt ggtggattgt taaggagagg gtggaaatgc atgaccgatg tgctgggagg
2521 tctgtggaaa tgtgtgacaa gagtgtgagt gtggaagtca gcgtctgcga aacaggcagc
2581 aacacagagg agtctgtgaa tgacctcaca ctcctcaaga caaacttgaa tctcaaagaa
2641 gtgcggtcta tcggttgtgg agattgttct gttgacgtga ccgtctgctc tccaaaggag
2701 tgcgcctccc ggggcgtgaa cactgaggct gttagccagg tggaagctgc cgtcatggca
2761 gtgcctcgta ctgcagacca ggacactagc acagatttgg aacaggtgca ccagttcacc
2821 aacaccgaga cggccaccct catagagtcc tgcaccaaca cttgtctaag cactttggac
2881 aagcagacca gcacccagac tgtggagacg cggacagtag ctgtaggaga aggccgtgtc
2941 aaggacatca actcctccac caagacgcgg tccattggtg ttggaacgtt gctttctggc
3001 cattctgggt ttgacaggcc atcagctgtg aagaccaaag agtcaggtgt ggggcagata
3061 aatattaacg acaactatct ggttggtctc aaaatgagga ctatagcttg tgggccacca
3121 cagttgactg tggggctgac agccagcaga aggagcgtgg gggttgggga tgaccctgta
3181 ggggaatctc tggagaaccc ccagcctcaa gctccacttg gaatgatgac tggcctggat
3241 cactacattg agcgtatcca gaagctgctg gcagaacagc agacactgct ggctgagaac
3301 tacagtgaac tggcagaagc tttcggggaa cctcactcac agatgggctc cctcaactct
3361 cagctcatca gcaccctgtc gtctatcaac tctgtcatga aatctgcaag cactgaagag
3421 ctgaggaacc ctgacttcca gaaaaccagt ctgggtaaaa tcacaggcaa ttatttggga
3481 tatacctgta agtgtggggg ccttcagtca ggaagtccct taagctccca gacatcccag
3541 cctgagcaag aagtggggac ctcagaagga aagccaatca gcagcctgga tgccttcccc
3601 actcaggaag gtacgctgtc tccagtgaac ctgacagacg accagatcgc cgctggcctc
3661 tatgcatgta caaacaatga aagtacactg aagtccatca tgaagaagaa agatggtaac
3721 aaagattcaa atggcgcaaa aaagaatctt cagtttgttg gcattaatgg agggtatgaa
3781 acaacttcaa gtgatgattc cagctcagat gaaagctctt cttccgagtc agatgacgag
3841 tgtgatgtca ttgagtatcc tcttgaagaa gaggaggagg aggaggatga agacactcgg
3901 ggaatggcag aagggcacca tgcagttaat attgaaggtt tgaagtctgc cagggtggaa
3961 gatgaaatgc aggttcaaga atgtgaacct gagaaggtgg aaatcagaga gaggtatgaa
4021 ttaagtgaaa agatgttgtc tgcatgcaac ttactgaaaa atactataaa tgaccccaaa
4081 gctttgacca gcaaagatat gaggttctgt ctgaacaccc tccagcacga gtggttccgc
4141 gtgtccagtc agaagtcagc cattccagcc atggtggggg actacatagc tgcttttgag
4201 gccatttccc cagatgtcct ccgctatgtc atcaacttgg cagacggcaa cggcaacaca
4261 gccctccatt acagcgtgtc ccactccaac ttcgagattg tgaagctgct gttagatgcc
4321 gatgtgtgta atgtggatca ccagaacaag gcaggctaca cccccatcat gttggcggcc
4381 ctcgccgctg tggaagcaga gaaggacatg cggattgtgg aagaactctt tggctgtggg
4441 gatgtgaatg ccaaagctag tcaggcggga cagacggccc tcatgctggc ggtcagtcac
4501 ggacggatag acatggtgaa gggccttctg gcctgtgggg ctgatgtcaa catccaggat
4561 gacgagggct ccacggccct catgtgtgcc agcgagcacg ggcacgtgga gattgtcaag
4621 ctgctgctgg cccagcccgg ctgcaacggt cacctagagg acaacgatgg cagcactgcg
4681 ctctcaatcg ccctggaagc aggacacaag gacatcgctg ttcttctgta tgcccatgtc
4741 aactttgcaa aagcccagtc tccgggcacc cctaggcttg gaaggaagac gtctcctggc
4801 cccacccacc gaggttcatt tgatggctcc gcgtcccgat caccggtcgc caccatggtg
4861 agcaagggcg aggagctgtt caccggggtg gtgcccatcc tggtcgagct ggacggcgac
4921 gtaaacggcc acaagttcag cgtgtccggc gagggcgagg gcgatgccac ctacggcaag
4981 ctgaccctga agttcatctg caccaccggc aagctgcccg tgccctggcc caccctcgtg
5041 accaccttga cctacggcgt gcagtgcttc gcccgctacc ccgaccacat gaagcagcac
5101 gacttcttca agtccgccat gcccgaaggc tacgtccagg agcgcaccat cttcttcaag
5161 gacgacggca actacaagac ccgcgccgag gtgaagttcg agggcgacac cctggtgaac

5221 cgcatcgagc tgaagggcat cgacttcaag gaggacggca acatcctggg gcacaagctg
5281 gagtacaact acaacagcca caaggtctat atcaccgccg acaagcagaa gaacggcatc
5341 aaggtgaact tcaagacccg ccacaacatc gaggacggca gcgtgcagct cgccgaccac
5401 taccagcaga acacccccat cggcgacggc cccgtgctgc tgcccgacaa ccactacctg
5461 agcacccagt ccaagctgag caaagacccc aacgagaagc gcgatcacat ggtcctgctg
5521 gagttcgtga ccgccgccgg gatcactctc ggcatggacg agctgtacaa gtaagcggcc
5581 gcgactctag atcataatca gccataccac atttgtagag gttttacttg ctttaaaaaa
5641 cctcccacac ctccccctga acctgaaaca taaaatgaat gcaattgttg ttgttaactt
5701 gtttattgca gcttataatg gttacaaata aagcaatagc atcacaaatt tcacaaataa
5761 agcatttttt tcactgcatt ctagttgtgg tttgtccaaa ctcatcaatg tatcttaagg
5821 cgtaaattgt aagcgttaat attttgttaa aattcgcgtt aaatttttgt taaatcagct
5881 cattttttaa ccaataggcc gaaatcggca aaatccctta taaatcaaaa gaatagaccg
5941 agatagggtt gagtgttgtt ccagtttgga acaagagtcc actattaaag aacgtggact
6001 ccaacgtcaa agggcgaaaa accgtctatc agggcgatgg cccactacgt gaaccatcac
6061 cctaatcaag ttttttgggg tcgaggtgcc gtaaagcact aaatcggaac cctaaaggga
6121 gcccccgatt tagagcttga cggggaaagc cggcgaacgt ggcgagaaag gaagggaaga
6181 aagcgaaagg agcgggcgct agggcgctgg caagtgtagc ggtcacgctg cgcgtaacca
6241 ccacacccgc cgcgcttaat gcgccgctac agggcgcgtc aggtggcact tttcggggaa
6301 atgtgcgcgg aacccctatt tgtttatttt tctaaataca ttcaaatatg tatccgctca
6361 tgagacaata accctgataa atgcttcaat aatattgaaa aaggaagagt cctgaggcgg
6421 aaagaaccag ctgtggaatg tgtgtcagtt agggtgtgga aagtccccag gctccccagc
6481 aggcagaagt atgcaaagca tgcatctcaa ttagtcagca accaggtgtg gaaagtcccc
6541 aggctcccca gcaggcagaa gtatgcaaag catgcatctc aattagtcag caaccatagt
6601 cccgccccta actccgccca tcccgcccct aactccgccc agttccgccc attctccgcc
6661 ccatggctga ctaatttttt ttatttatgc agaggccgag gccgcctcgg cctctgagct
6721 attccagaag tagtgaggag gcttttttgg aggcctaggc ttttgcaaag atcgatcaag
6781 agacaggatg aggatcgttt cgcatgattg aacaagatgg attgcacgca ggttctccgg
6841 ccgcttgggt ggagaggcta ttcggctatg actgggcaca acagacaatc ggctgctctg
6901 atgccgccgt gttccggctg tcagcgcagg ggcgcccggt tctttttgtc aagaccgacc
6961 tgtccggtgc cctgaatgaa ctgcaagacg aggcagcgcg gctatcgtgg ctggccacga
7021 cgggcgttcc ttgcgcagct gtgctcgacg ttgtcactga agcgggaagg gactggctgc
7081 tattgggcga agtgccgggg caggatctcc tgtcatctca ccttgctcct gccgagaaag
7141 tatccatcat ggctgatgca atgcggcggc tgcatacgct tgatccggct acctgcccat
7201 tcgaccacca agcgaaacat cgcatcgagc gagcacgtac tcggatggaa gccggtcttg
7261 tcgatcagga tgatctggac gaagagcatc aggggctcgc gccagccgaa ctgttcgcca
7321 ggctcaaggc gagcatgccc gacggcgagg atctcgtcgt gacccatggc gatgcctgct
7381 tgccgaatat catggtggaa aatggccgct tttctggatt catcgactgt ggccggctgg
7441 gtgtggcgga ccgctatcag gacatagcgt tggctacccg tgatattgct gaagagcttg
7501 gcggcgaatg ggctgaccgc ttcctcgtgc tttacggtat cgccgctccc gattcgcagc
7561 gcatcgcctt ctatcgcctt cttgacgagt tcttctgagc gggactctgg ggttcgaaat
7621 gaccgaccaa gcgacgccca acctgccatc acgagatttc gattccaccg ccgccttcta
7681 tgaaaggttg ggcttcggaa tcgttttccg ggacgccggc tggatgatcc tccagcgcgg
7741 ggatctcatg ctggagttct tcgcccaccc taggggggagg ctaactgaaa cacggaagga
7801 gacaataccg gaaggaaccc gcgctatgac ggcaataaaa agacagaata aaacgcacgg
7861 tgttgggtcg tttgttcata aacgcggggt tcggtcccag ggctggcact ctgtcgatac
7921 cccaccgaga ccccattggg gccaatacgc ccgcgtttct tccttttccc caccccaccc
7981 cccaagttcg ggtgaaggcc cagggctcgc agccaacgtc ggggcggcag gccctgccat
8041 agcctcaggt tactcatata tactttagat tgatttaaaa cttcattttt aatttaaaag
8101 gatctaggtg aagatccttt ttgataatct catgaccaaa atcccttaac gtgagttttc
8161 gttccactga gcgtcagacc ccgtagaaaa gatcaaagga tcttcttgag atcctttttt
8221 tctgcgcgta atctgctgct tgcaaacaaa aaaaccaccg ctaccagcgg tggtttgttt
8281 gccggatcaa gagctaccaa ctctttttcc gaaggtaact ggcttcagca gagcgcagat
8341 accaaatact gtccttctag tgtagccgta gttaggccac cacttcaaga actctgtagc
8401 accgcctaca tacctcgctc tgctaatcct gttaccagtg gctgctgcca gtggcgataa
8461 gtcgtgtctt accgggttgg actcaagacg atagttaccg gataaggcgc agcggtcggg
8521 ctgaacgggg ggttcgtgca cacagcccag cttggagcga acgacctaca ccgaactgag
8581 atacctacag cgtgagctat gagaaagcgc cacgcttccc gaagggagaa aggcggacag
8641 gtatccggta agcggcaggg tcggaacagg agagcgcacg agggagcttc caggggggaaa
8701 cgcctggtat ctttatagtc ctgtcgggtt tcgccacctc tgacttgagc gtcgattttt
8761 gtgatgctcg tcagggggggc ggagcctatg gaaaaacgcc agcaacgcgg cctttttacg
8821 gttcctggcc ttttgctggc cttttgctca catgttcttt cctgcgttat cccctgattc

```
      8881 tgtggataac cgtattaccg ccatgcat
//
```

Appendix references

Amano M, Ito M, Kimura K, Fukata Y, Chihara K, Nakano T, Matsuura Y, Kaibuchi K. Phosphorylation and activation of myosin by Rho-associated kinase (Rho-kinase) J Biol Chem. 1996;271:20246–20249.

Alfonso Bolado-Carrancio, Oleksii S Rukhlenko, Elena Nikonova, Mikhail A Tsyganov, Anne Wheeler, Amaya Garcia-Munoz, Walter Kolch, Alex von Kriegsheim, Boris N Kholodenko. Periodic propagating waves coordinate RhoGTPase network dynamics at the leading and trailing edges during cell migration. Elife. 2020 Jul 24;9:e58165. J Cell Biol. 2019 Sep 2;218(9):3077-3097.

Mihai L Azoitei, Jungsik Noh, Daniel J Marston, Philippe Roudot, Christopher B Marshall, Timothy A Daugird, Sidney L Lisanza, María-José Sandí, Mitsu Ikura, John Sondek, Robert Rottapel, Klaus M Hahn, Gaudenz Danuser. Spatiotemporal dynamics of GEF-H1 activation controlled by microtubule- and Src-mediated pathways. J Cell Biol. 2019 218(9):3077-3097.

Benjamin P Bouchet, Rosemarie E Gough, York-Christoph Ammon, Dieudonnée van de Willige, Harm Post, Guillaume Jacquemet, AF Maarten Altelaar, Albert JR Heck, Benjamin T Goult, Anna Akhmanova (2016) Talin-KANK1 interaction controls the recruitment of cortical microtubule stabilizing complexes to focal adhesions eLife 5:e18124.

A Efimov, I Kaverina. Significance of microtubule catastrophes at focal adhesion sites. Cell Adh Migr. 2009 3(3): 285–287.

Mira Krendel, Frank T. Zenke & Gary M. Bokoch. Nucleotide exchange factor GEF-H1 mediates cross-talk between microtubules and the actin cytoskeleton. Nature Cell Biology volume 4, pages294–301 (2002)

Shailaja Seetharaman, Benoit Vianay, Vanessa Roca, Aaron J Farrugia, Chiara De Pascalis, Batiste Boëda, Florent Dingli, Damarys Loew, Stéphane Vassilopoulos, Alexander Bershadsky, Manuel Théry, Sandrine Etienne-Manneville. Microtubules tune mechanosensitive cell responses. Nat Mater. 2022 21(3):366-377.

W. A. Strauss. Partial Differential Equations: An Introduction. Wiley; 2nd edition (2007)

Zhiqi Sun, Hui-Yuan Tseng, Steven Tan, Fabrice Senger, Laetitia Kurzawa, Dirk Dedden, Naoko Mizuno, Anita A Wasik, Manuel Thery, Alexander R. Dunn, and Reinhard Fässler. Kank2 activates talin, reduces force transduction across integrins and induces central adhesion formation. Nat Cell Biol. 2016 18(9): 941–953.

Jiping Yue, Min Xie, Xuewen Gou, Philbert Lee, Michael D Schneider, Xiaoyang Wu. Microtubules regulate focal adhesion dynamics through MAP4K4. Dev Cell. 2014 31(5):572-85.
