# Supplementary material for: Focal adhesions are controlled by microtubules through local contractility regulation
Source: EMBO J. 2024 May 20;43(13):9. doi: 10.1038/s44318-024-00114-4 (PMC11217342; doi:10.1038/s44318-024-00114-4)
Supplement: Supplementary file 2 — Movie EV1 [file 44318_2024_114_MOESM2_ESM.zip › Legend movie EV1.docx]

**Movie EV1**

**OptoKANK activation promotes focal adhesion sliding and disassembly**

HT1080 cell transfected with OptoKANK (KN + ΔKN) and vinculin-mIFP was illuminated (488 nm) over the focal adhesion; approximate area of illumination is demarcated by the blue circle. Typical movie in which the focal adhesions labeled by vinculin-mIFP slide and disassemble upon OptoKANK activation. Acquisition rate is 1 frame/5 sec and display rate is 30 frames/sec.
