# Supplementary material for: Focal adhesions are controlled by microtubules through local contractility regulation
Source: EMBO J. 2024 May 20;43(13):9. doi: 10.1038/s44318-024-00114-4 (PMC11217342; doi:10.1038/s44318-024-00114-4)
Supplement: Supplementary file 3 — Movie EV2 [file 44318_2024_114_MOESM3_ESM.zip › Legend movie EV2.docx]

**Movie EV2**

**Nodazole treatment prevents focal adhesion disassembly upon OptoKANK activation**

HT1080 cell transfected with OptoKANK (KN + ΔKN) and vinculin-mIFP was illuminated (488 nm) over the focal adhesion; approximate area of illumination is demarcated by the blue circle. The cell was treated with nocodazole (1µM) for 3 hours prior illumination and imaging of the focal adhesion labeled by vinculin-mIFP. Note that in the nocodazole treated cell, the focal adhesion remains unchanged after OptoKANK activation. Acquisition rate is 1 frame/5 sec and display rate is 30 frames/sec.
