# Supplementary material for: Focal adhesions are controlled by microtubules through local contractility regulation
Source: EMBO J. 2024 May 20;43(13):9. doi: 10.1038/s44318-024-00114-4 (PMC11217342; doi:10.1038/s44318-024-00114-4)
Supplement: Supplementary file 4 — Movie EV3 [file 44318_2024_114_MOESM4_ESM.zip › Legend movie EV3.docx]

**Movie EV3**

**Nocodazole washout rescues focal adhesion sliding and disassembly upon OptoKANK activation**

HT1080 cell transfected with OptoKANK (KN + ΔKN) and vinculin-mIFP was illuminated (488 nm) over the focal adhesion (in the blue circle). The cell was treated with nocodazole (1µM) for 3 hours followed by 2 hours washout prior illumination and imaging of the focal adhesion labeled by vinculin-mIFP. A typical movie in which the focal adhesion slides and disassembles upon OptoKANK activation. Acquisition rate is 1 frame/5 sec and display rate is 30 frames/sec.
