# Supplementary material for: Focal adhesions are controlled by microtubules through local contractility regulation
Source: EMBO J. 2024 May 20;43(13):9. doi: 10.1038/s44318-024-00114-4 (PMC11217342; doi:10.1038/s44318-024-00114-4)
Supplement: Supplementary file 5 — Movie EV4 [file 44318_2024_114_MOESM5_ESM.zip › Legend movie EV4.docx]

**Movie EV4**

**OptoKANK activation promotes microtubule tips targeting to focal adhesion**

HT1080 cell transfected with OptoKANK (KN + ΔKN) and EB3-mIFP was illuminated (488 nm) over the focal adhesions (in the blue circles) and the EB3 comets (left panel) were tracked automatically using the plusTipTracker software(Applegate *et al.*, 2011; Matov *et al.*, 2010) (right panel). Non illuminated focal adhesion areas (white circles on left panel and dark circles on right panel) were used as control. Acquisition rate is 1 frame/3 sec and display rate is 20 frames/sec.
