# Supplementary material for: Focal adhesions are controlled by microtubules through local contractility regulation
Source: EMBO J. 2024 May 20;43(13):9. doi: 10.1038/s44318-024-00114-4 (PMC11217342; doi:10.1038/s44318-024-00114-4)
Supplement: Supplementary file 6 — Movie EV5 [file 44318_2024_114_MOESM6_ESM.zip › Legend movie EV5.docx]

**Movie EV5**

**Withdrawal of OptoKANK-targeted microtubules from focal adhesion.**

HT1080 cell transfected with OptoKANK (KN + ΔKN) and treated with SiR-Tubulin (250 nM for 3 hours) observed with a focus on focal adhesion visualized by mApple-KN-LOV2ssrA (green). The movie started after onset of illumination activating OptoKANK. Note that microtubules (magenta) overlapping with the outlined focal adhesion area (yellow), underwent rapid withdrawal and finally disappeared from the focal adhesion area. Acquisition rate is 1 frame/5 sec and display rate is 15 frames/sec.
