# Supplementary material for: Focal adhesions are controlled by microtubules through local contractility regulation
Source: EMBO J. 2024 May 20;43(13):9. doi: 10.1038/s44318-024-00114-4 (PMC11217342; doi:10.1038/s44318-024-00114-4)
Supplement: Supplementary file 7 — Movie EV6 [file 44318_2024_114_MOESM7_ESM.zip › Legend movie EV6.docx]

**Movie EV6**

**OptoKANK activation results in myosin-II filaments accumulation in the vicinity of the illuminated focal adhesion proximal end.**

HT1080 cell transfected with OptoKANK (KN + ΔKN) and MLC-mIFP was illuminated (488 nm) over the focal adhesion (yellow circle). The position of focal adhesion is indicated in the first frame by KN-mApple fluorescence. Accumulation of myosin-II filaments based on the MLC-mIFP intensity appears in centripetal direction from focal adhesions following the onset of illumination (see white arrow). Acquisition rate is 1 frame/5 sec and display rate is 10 frames/sec.
