# Supplementary material for: Focal adhesions are controlled by microtubules through local contractility regulation
Source: EMBO J. 2024 May 20;43(13):9. doi: 10.1038/s44318-024-00114-4 (PMC11217342; doi:10.1038/s44318-024-00114-4)
Supplement: Supplementary file 8 — Movie EV7 [file 44318_2024_114_MOESM8_ESM.zip › Legend movie EV7.docx]

**Movie EV7**

**OptoKANK activation results in actin accumulation in the vicinity of the illuminated focal adhesion proximal end.**

HT1080 cell transfected with OptoKANK (KN + ΔKN) and pre-incubated with SiR-actin for 1h was illuminated (488 nm) over the focal adhesion (yellow circle). The focal adhesion is visualized using KN-mApple fluorescence. Accumulation of actin filaments visualized by the SiR-actin labeling appears in centripetal direction from focal adhesions following the onset of illumination. Acquisition rate is 1 frame/5 sec and display rate is 30 frames/sec.
