# Supplementary material for: Focal adhesions are controlled by microtubules through local contractility regulation
Source: EMBO J. 2024 May 20;43(13):9. doi: 10.1038/s44318-024-00114-4 (PMC11217342; doi:10.1038/s44318-024-00114-4)
Supplement: Supplementary file 9 — Movie EV8 [file 44318_2024_114_MOESM9_ESM.zip › Legend movie EV8.docx]

**Movie EV8**

**GEF-H1 knockdown prevents focal adhesion disassembly upon OptoKANK activation**

HT1080 cells transfected with OptoKANK (KN + ΔKN) and vinculin-mIFP was illuminated (488 nm) over the focal adhesion (blue circle) in control (left panel) or after GEF-H1 knockdown (right panel). While the focal adhesion slides and disassembles upon OptoKANK activation in ctrl, depletion of GEF-H1 typically prevents the sliding and disassembly of focal adhesion. Acquisition rate is 1 frame/5 sec and display rate is 20 frames/sec.
