# Supplementary material for: Focal adhesions are controlled by microtubules through local contractility regulation
Source: EMBO J. 2024 May 20;43(13):9. doi: 10.1038/s44318-024-00114-4 (PMC11217342; doi:10.1038/s44318-024-00114-4)
Supplement: Supplementary file 10 — Movie EV9 [file 44318_2024_114_MOESM10_ESM.zip › Legend movie EV9.docx]

**Movie EV9**

**Rescuing the GEF-H1 knockdown effect on focal adhesion disassembly upon OptoKANK activation by activation of RhoA with CNO3**

GEF-H1-knocked down HT1080 cell transfected with OptoKANK (KN + ΔKN) and vinculin-mIFP was illuminated (488 nm) over the focal adhesion (yellow circle). CNO3 (1 µg/ml) was added 2h prior the onset of illumination. Upon OptoKANK activation, the focal adhesion slides and disassembles in spite of GEF-H1 depletion. Acquisition rate is 1 frame/5 sec and display rate is 20 frames/sec.
