# Supplementary material for: Focal adhesions are controlled by microtubules through local contractility regulation
Source: EMBO J. 2024 May 20;43(13):9. doi: 10.1038/s44318-024-00114-4 (PMC11217342; doi:10.1038/s44318-024-00114-4)
Supplement: Supplementary file 11 — Movie EV10 [file 44318_2024_114_MOESM11_ESM.zip › Legend movie EV10.docx]

**Movie EV10**

**Inhibition of endocytosis results in local membrane bleb formation upon OptoKANK activation but does not protect focal adhesion**

HT1080 cells transfected with OptoKANK (KN + ΔKN) and vinculin-mIFP was illuminated (488 nm) over the focal adhesion (yellow circle) in control (left panel) or in the presence of the endocytosis inhibitor, dynasore (right panel). OptoKANK activation of cell treated with dynasore results in formation of membrane bleb at the illuminated sites (phase contrast) and induces sliding and disassembly of focal adhesion as visualized by vinculin-mIFP (red). Acquisition rate is 1 frame/5 sec and display rate is 20 frames/sec.
