# Supplementary material for: Focal adhesions are controlled by microtubules through local contractility regulation
Source: EMBO J. 2024 May 20;43(13):9. doi: 10.1038/s44318-024-00114-4 (PMC11217342; doi:10.1038/s44318-024-00114-4)
Supplement: Supplementary file 13 — Expanded View Figures [file 44318_2024_114_MOESM13_ESM.pdf]

Expanded View Figure

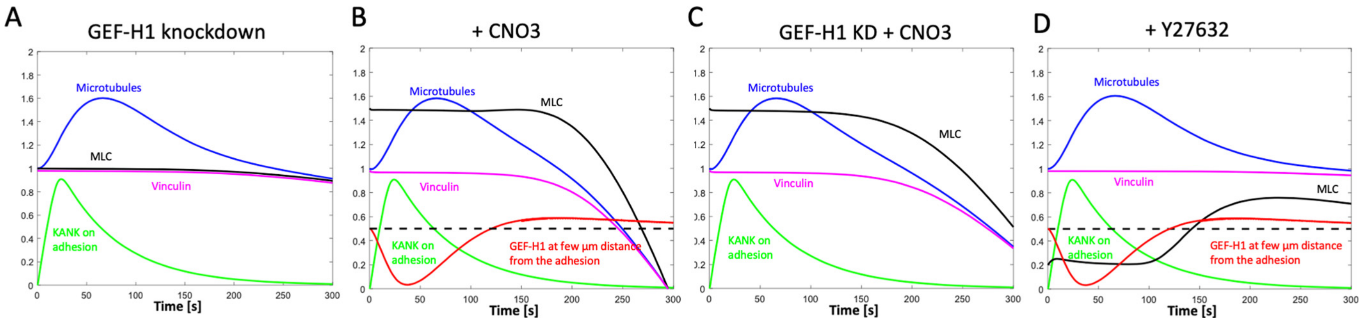

Figure EV1. Model-predicted time series for microtubule, vinculin, KANK, GEF-H1 and myosin densities upon simulated perturbations.

The following perturbations: GEF-H1 knockdown (**A**), treatment with CNO3 (**B**), GEF-H1 knockdown + CNO3 (**C**) and treatment with Y27632 (**D**) have been simulated based on model parameters described in the Appendix. Dashed line indicates the threshold above which GEF H1 significantly affects the myosin activation pathway.

 © The Author(s)
